# Supplementary material for: Universal Structural Influence on the 2D Electron Gas at SrTiO3 Surfaces
Source: Adv Sci (Weinh). 2021 Sep 17;8(22):2100602. doi: 10.1002/advs.202100602 (PMC8596100; doi:10.1002/advs.202100602)
Supplement: Supplementary file 1 — Supporting Information [file ADVS-8-2100602-s001.pdf]

© 2021 Wiley-VCH GmbH

ADVANCED SCIENCE
Open Access

Supporting Information

for *Adv. Sci.,* DOI: 10.1002/advs.202100602

Universal Structural Influence on the 2D Electron Gas at $SrTiO_3$ Surfaces

Eduardo B. Guedes, Stefan Mu, W. H. Brito, Marco Caputo, Hang Li, Nicholas C. Plumb, J. Hugo Dil, and Milan Radovic**

Supporting Information for "Universal Structural Influence on the 2D Electron Gas at $SrTiO_3$ Surfaces"

Eduardo B. Guedes,[1,2] Stefan Muff,[1,2] W. H. Brito,[3] Marco Caputo,[1,4]
Hang Li,[5,1] Nicholas C. Plumb,[1] J. Hugo Dil*,[2,1] and Milan Radović*[1]

[1] *Photon Science Division, Paul Scherrer Institut, CH-5232 Villigen, Switzerland*
[2] *Institut de Physique, École Polytechnique Fédérale de Lausanne, CH-1015 Lausanne, Switzerland*
[3] *Departamento de Física, Universidade Federal de Minas Gerais, C.P. 702, 30123-970 Belo Horizonte, Minas Gerais, Brazil*
[4] *Elettra Sincrotrone Trieste, s.s. 14 km 163.5 in Area Science Park, 34149 Trieste, Italy*
[5] *Department of Energy Conversion and Storage, Technical University of Denmark, 2800 Kgs. Lyngby, Denmark*

(Dated: June 23, 2021)

I. FLAT STO

A. Resistivity

Figure S1 shows the sheet resistance of Nb:STO crystals, measured with the standard four-point probe setup. From 8 to ∼ 80 K, the sample shows a metallic behavior. Close to the bulk structural phase transition the conductivity suddenly changes and becomes more insulating. At around 110 K, the sample turns metallic again, up to room temperature. This behavior may be the reason for the jump in E_F and its subsequent recovery seen in Figures 1a and 1b of the main text.

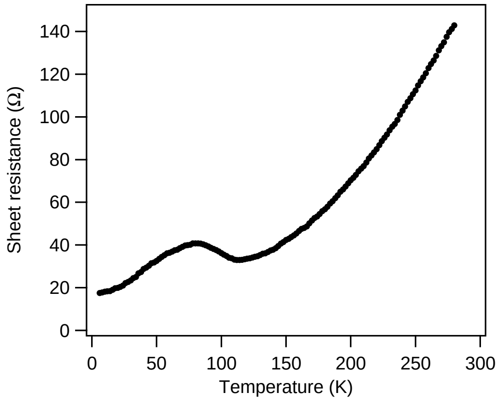

FIG. S1. Sheet resistance of a flat Nb:STO sample treated as described in the Methods session of the main text.

B. Temperature ramp

The slow temperature ramp (∼0.1 K/min) displayed in Figure S2 was recorded with two diodes, one in contact with the cryostat (blue) and the other in close proximity to the sample (orange). The arrow indicates the start of the temperature ramp. Although the temperature ramps were performed very slowly, the error in the temperature reading is expected to be around 7%. This is partly due to the distance between the sample diode and the sample surface and partly due to the thermal lag between the sample and this diode, similar to the thermal lag between the sample diode and the cryostat illustrated by the offset between the orange and blue curves.

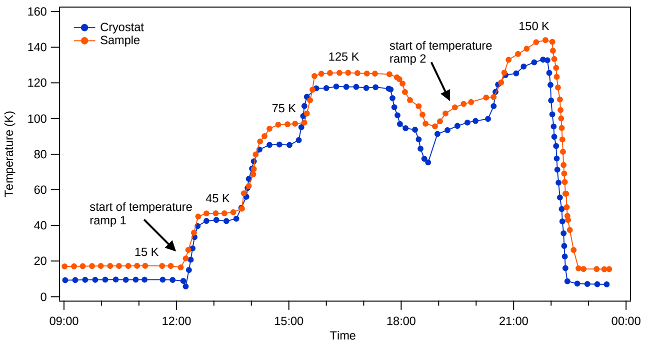

FIG. S2. Temperature evolution during the experiment with the flat STO crystal. The blue curve indicates the temperature of the cryostat and the orange curve the temperature of the diode closest to the sample.

C. Additional band dispersion maps

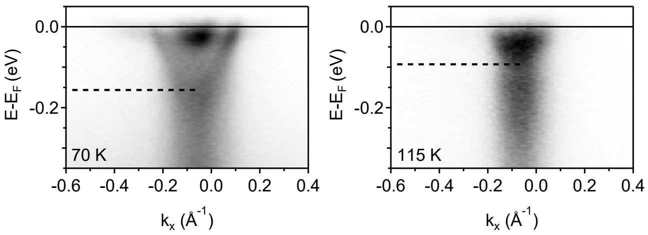

FIG. S3. Band dispersion maps acquired at 70 and 115 K for flat STO. The dashed line indicates the bottoms of the d_{xy} band.

Figure S3 shows the band dispersion maps acquired for the flat STO samples with hν=85 eV, with C^+ polarization around the $\overline{\Gamma_{10}}$ point at 70 and 115 K. The dashed lines indicate the band bottoms at 141 and 91 meV, respectively. These values were used in Figure 5(c) of the main text, along with the information from the other band maps in Figure 1 of the main text.

D. Compositional analysis

The samples were obtained nominally TiO_2-terminated from the supplier, after etching, as described in the sample preparation and further treated in-situ with annealing in 100 mbar of O_2 at 550 °C. This treatment, along with others shown in [S1], is a standard method to clean $SrTiO_3$ and create the 2DEG. In order to understand the effect of these treatments, we performed XPS measurements on unetched and etched $SrTiO_3$ samples, both of them non-annealed. In Figure S4 we compare these measurements with XPS data from the etched+annealed sample shown in the main text. Using the integrated intensity from the Sr 3d and Ti 3p core levels, the calculated Sr/Ti ratios are 1.14, 0.76 and 1.41 for the unetched, etched, and etched+annealed (reported in the manuscript) samples. With respect to the data shown in the supplementary information of [S2], these values fall in the categories "as-received STO wafer ($\sim$ 1.18)", "TiO2-term. ($\sim$ 0.8)", and "SrO-term. ($\sim$ 1.5)" respectively. We thus propose that the excess of Sr on the surface is due to the etching process in conjunction with the last annealing step.

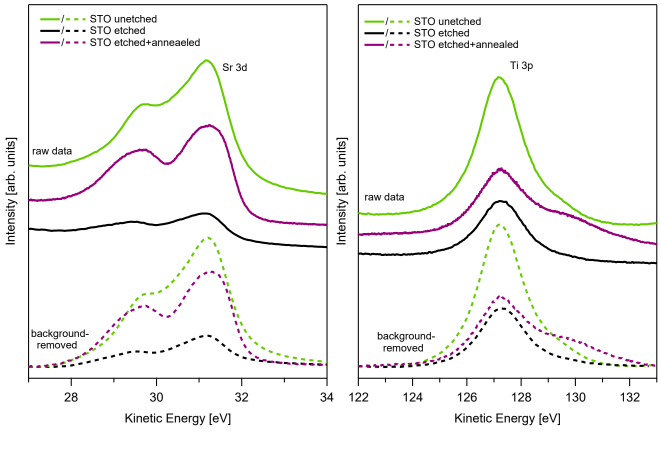

FIG. S4. Comparison of XPS spectra of unetched, etched and etched+annealed samples.

II. VICINAL STO SURFACES

Figure S5 shows the LEED pattern recorded for the 5°- and 10°-miscut STO samples, where we observe a (1×1) surface. The vertical streaking around the main spots in due to the step potential and is typical for stepped surfaces. For the 10°-miscut surface the smaller step distance results in a better separation of the diffraction spots, although a detailed analysis would require a dedicated LEED set-up [S3]. Of main importance to the current work is that the average step density is maintained after sample preparation.

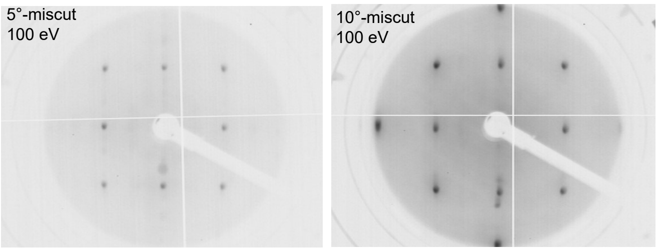

FIG. S5. LEED patterns of the 5°- and 10°-miscut STO samples showing no evident sign of surface reconstructions.

No signature of the steps, nor of a surface reconstruction is observed in the Fermi surface in the k_x-k_z plane, shown in Figures ?? and Figures ??. As in the main text, an inner potential V_0=14.5 eV was used in the conversion from $h\nu$ to k_z, and the the straight lines around the zone centres correspond to the non-dispersive, 2D light bands with d_{xy} character. A similar conclusion can be drawn by considering the Fermi surfaces in the k_x-k_y plane, shown in Figures S8 and S9 spanning several Brillouin Zones. This data which resembles the Fermi surface from the flat STO sample, but with a smaller band filling.

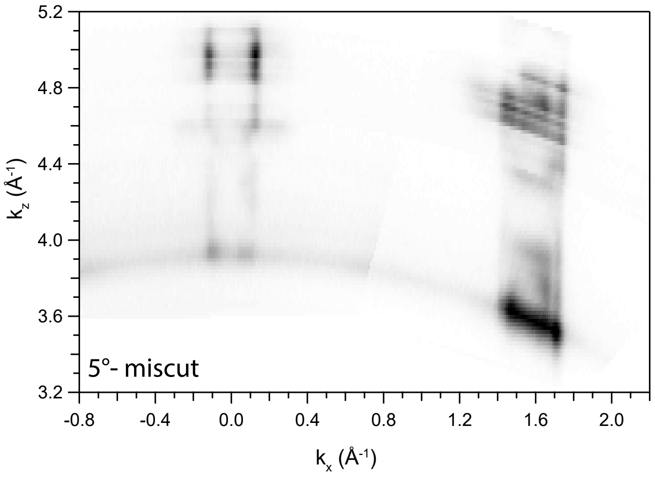

FIG. S6. Fermi surface in the k_z-k_x plane of the 5°-miscut STO samples.

Figure S10 shows details of the 2DEG formed on the 5°-miscut sample around the $\overline{\Gamma_{10}}$ at 20 K. The top panels show the Fermi surface obtained using hν=85 eV LV- and LH-polarized light respectively, in which the dashed lines indicates where the band dispersion maps in the lower panels were measured. In turn, the dashed lines in the

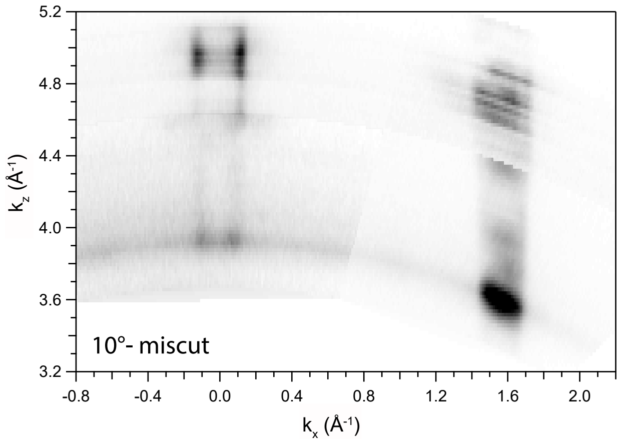

FIG. S7. Fermi surface in the k_z-k_x plane of the 10°-miscut STO samples.

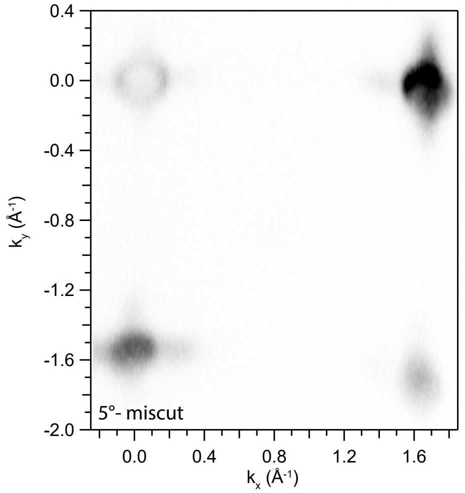

FIG. S8. Fermi surface maps in the k_x-k_y plane of the 5°-miscut STO surfaces.

band dispersion maps indicate the bottom of the d_{xy} and d_{yz} bands, which were used in Figure 5(c) of the main text.

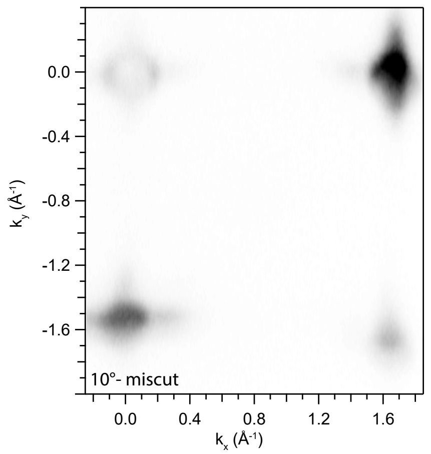

FIG. S9. Fermi surface maps in the k_x-k_y plane of the 10°-miscut STO surfaces.

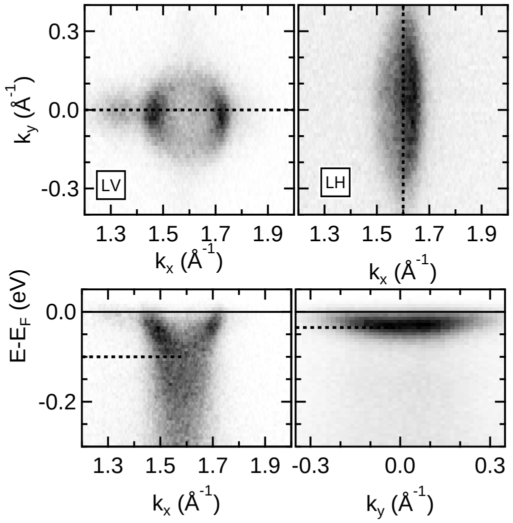

FIG. S10. Fermi surface band dispersion maps of the 5°-miscut STO sample measured with LV- and LH-polarized light.

III. AB INITIO CALCULATIONS

Figure S11 shows the calculated band structure highlighting the contributions from the $3d_{xy,yz,xz}$ originating from Ti atoms localized in either the bulk or the surface layer. As mentioned in the main text, this band structure reveals an electron-like band with Ti-d_{xy} character almost entirely originating from the surface layer, as well as the d_{yz} d_{xz} bands with mixed bulk-surface origin, in good agreement with previous works [S1].

In our slab calculations, the dispersion of conduction band in the direction perpendicular to the slab surface is replaced by two kinds of states. The first are a series of bands centered at different energies and composed from $3d$ orbitals from all Ti atoms, with their relative

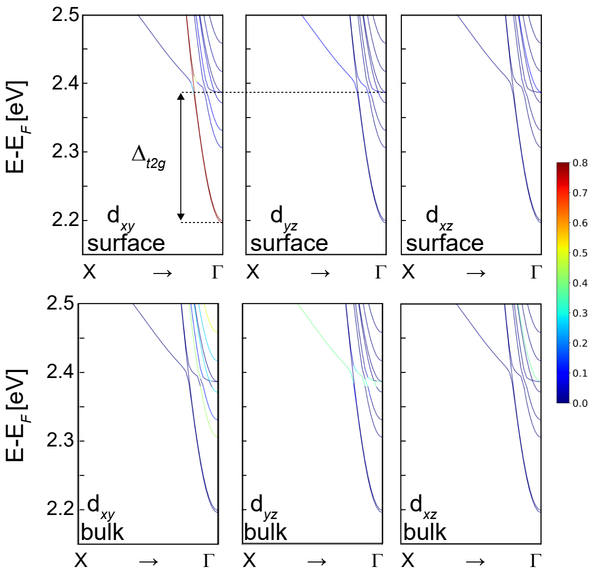

FIG. S11. Band structure of the SrO-terminated slab highlighting the surface- and bulk-projected d$_{xy}$, d$_{xz}$ and d$_{yz}$ orbital character of the bands.

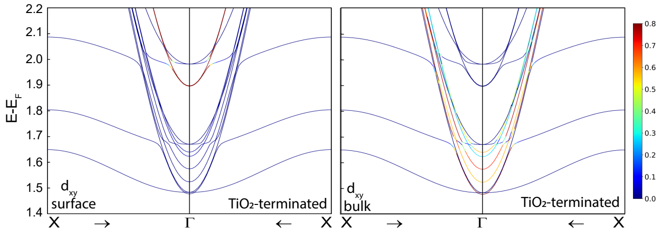

FIG. S12. Band structure of the TiO_2-terminated slab highlighting the d$_{xy}$ orbital character from bulk and surface layers.

contributions depending on each respective atomic layer. These states are greatly affected by the slab thickness and form the bulk bands in the limit of infinite unit cells. The second kind are states strongly localized in the surface layer, which are only weakly subject finite-size effects. Our surface-derived d$_{xy}$ states show a relatively small breaking of degeneracy of around 3 meV, which we assign to the finite size of the slab [S4].

As pointed out in the main text, we have also performed DFT calculations for a TiO_2-terminated slab, whose band structure is shown on Figure S12, highlighting the contributions from the bulk and surface Ti 3d_{xy} orbitals. The calculation shows that, in contrast with the SrO-terminated slab presented in Figure 5 of the main text, the lowest-lying bands have bulk character, and therefore are in direct contrast with surface character of the 2DEG.

Due to its symmetry, our cubic slab does not allow atomic displacements in the xy plane. To check whether this has an effect on the results, we also calculated a 1×1×3 STO slab in the AFD phase (please note that the unit cell is doubled with respect to the bulk one),

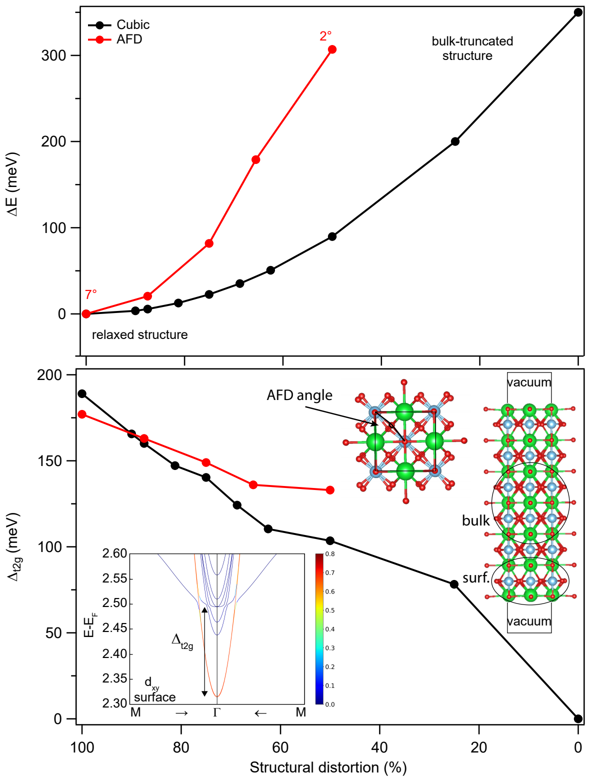

FIG. S13. Relative total energies calculated for each respective degree of distortion (relative to each relaxed structure) and value of Δ_{t2g}, obtained for SrO-terminated, both cubic and AFD structures. The insets show details of the relaxed AFD slab and its band structure, highlighting Δ_{t2g}.

which allows for such displacements. In the top panel of Figure S13 we show the total energies calculated for each respective degree of distortion, with respect to the relaxed structure, for SrO-terminated slabs in the cubic and AFD phases. In particular for the AFD calculations were performed for different AFD angles, ranging from 2 to 7°. In the bottom panel of Figure S13 we show the AFD structure and highlight the AFD angle at the surface layer. The atoms of the central layer are fixed (bulk), while the ones at the outer unit cells (surface) are allowed to move. In this structure, the atoms are allowed to move in the xy-plane as well as in the z-direction. The inset further shows the the resulting band structure calculated for the relaxed structure (AFD angle of 7°) is shown in the bottom inset of Figure S13, indicating the splitting Δ_{t2g}. Please note that the AFD structure is rotated 45°in-plane with respect to the cubic structure, which is why we show the band structure plotted in the ΓM direction in this case. In these calculations, due to limitations of our supercell size, there is a tiny lift of degeneracy regarding the surface d$_{xy}$ band of 1.5 meV. The main panel of Figure S13 compares the value of Δ_{t2g} obtained for both structures for different degrees of distortion, where we observe a similar trend.

[S1] N. C. Plumb, M. Salluzzo, E. Razzoli, M. Månsson, M. Falub, J. Krempasky, C. E. Matt, J. Chang, M. Schulte, J. Braun, H. Ebert, J. Minár, B. Delley, K.-J. Zhou, T. Schmitt, M. Shi, J. Mesot, L. Patthey, and M. Radović, Mixed dimensionality of confined conducting electrons in the surface region of $SrTiO_3$, Phys. Rev. Lett. **113**, 086801 (2014).

[S2] S. N. Rebec, T. Jia, H. M. Sohail, M. Hashimoto, D. Lu, Z.-X. Shen, and R. G. Moore, Dichotomy of the photo-induced 2-dimensional electron gas on srtio3 surface terminations, Proceedings of the National Academy of Sciences **116**, 16687 (2019), https://www.pnas.org/content/116/34/16687.full.pdf.

[S3] C. Brand, H. Pfnür, G. Landolt, S. Muff, J. H. Dil, T. Das, and C. Tegenkamp, Observation of correlated spin-orbit order in a strongly anisotropic quantum wire system, Nature Communications **6**, 8118 (2015).

[S4] S. S. Ghosh and E. Manousakis, Structure and ferromagnetic instability of the oxygen-deficient $srtio_3$ surface, Phys. Rev. B **94**, 085141 (2016).
